# Exercise With Low-Loads and Concurrent Partial Blood Flow Restriction Combined With Patient Education in Females Suffering From Gluteal Tendinopathy: A Feasibility Study

**DOI:** 10.3389/fspor.2022.881054

**Published:** 2022-04-14

**Authors:** Mathias Høgsholt, Stian Langgård Jørgensen, Nanna Rolving, Inger Mechlenburg, Lisa Urup Tønning, Marie Bagger Bohn

**Affiliations:** ^1^Department of Occupational and Physical Therapy, Horsens Regional Hospital, Horsens, Denmark; ^2^H-HIP, Department of Orthopedic Surgery, Horsens Regional Hospital, Horsens, Denmark; ^3^Department of Clinical Medicine, Aarhus University, Aarhus, Denmark; ^4^Center of Rehabilitation Research, DEFACTUM, Central Denmark Region, Aarhus, Denmark; ^5^Department of Physical and Occupational Therapy, Aarhus University Hospital, Aarhus, Denmark; ^6^Department of Orthopedic Surgery, Aarhus University Hospital, Aarhus, Denmark

**Keywords:** blood flow restriction, feasibility, patient education, venous occlusion, exercise therapy, gluteal tendinopathy

## Abstract

**Introduction:**

To date, there exists no gold standard conservative treatment for lateral hip pain due to tendinopathy of the gluteus medius and/or minimus tendon (GT), a condition often complicated by pain and disability. Higher loads during everyday activities and exercise seems to be contraindicated with GT. The purpose of this study was to evaluate the feasibility of exercise with low-loads concurrent partial blood flow restriction (LL-BFR) and patient education for patients present GT.

**Methods:**

Recruitment took place at three hospitals in the Central Denmark Region. The intervention consisted of daily sessions for 8 weeks with one weekly supervised session. From week three patients exercised with applied partial blood flow restriction by means of a pneumatic cuff around the proximal thigh of the affected leg. Throughout the intervention patients received patient education on their hip condition. Sociodemographic and clinical variables were collected at baseline. The feasibility of LL-BFR was conducted by adherence to the exercise protocol and drop-out rate. Patient reported outcome measures (The Victorian Institute of Sport Assessment-Gluteal Questionnaire, EuroQol - 5 Dimensions-Visual Analogue Scale, Oxford Hip Score, Copenhagen Hip and Groin Outcome Score), maximal voluntary isometric hip abduction-, hip extension, and knee extension strength (Nm/kg) measured using a handheld dynamometer, and functional capacity tests (30 second chair-stand test and a stair-climb test) was conducted as secondary outcomes.

**Results:**

Sixteen women with a median (IQR) age of 51 (46–60) years were included. Median (IQR) Body Mass Index was 26.69 (23.59–30.46) kg/m2. Adherence to the total number of training sessions and the LL-BFR was 96.4 and 94.4%, respectively. Two patients dropped out due to (i) illness before initiation of LL-BFR and (ii) pain in the affected leg related to the LL-BFR-exercise. At follow-up both pain levels and patient-reported outcome measures improved. Isometric hip abduction-, hip extension-, and knee extension strength on both legs and functional performance increased. Conclusion: LL-BFR-exercise seems feasible for treatment of GT. At follow-up, a high adherence and low drop-out rate were observed. Further, patients reported clinically relevant reductions in pain, and showed significant increases in isometric hip and knee strength.

## Introduction

Gluteal tendinopathy (GT) of the hip abductor muscle tendons (gluteus medius and minimus) has recently been recognized as the primary underlying pathology causing greater trochanteric pain syndrome (Kagan, 1999; Kingzett-Taylor et al., 1999; Bird et al., 2001; Kong et al., 2007; Fearon et al., 2010; Grimaldi and Fearon, 2015). The patient population primarily consists of females aged 40–60 years (Grimaldi et al., 2015). Recent studies indicate that GT is among the most prevalent lower limb tendinopathies in adults seen in general practice and is associated with moderate to severe hip-related pain and disability (Fearon et al., 2014; Albers et al., 2016; Riel et al., 2019; Bohn et al., 2021). GT presents pain directly above the greater trochanter (Grimaldi and Fearon, 2015; Speers and Bhogal, 2017). Further, stair climbing, sleeping on the symptomatic side, and walking have been reported by patients with GT to aggravate pain (Woodley et al., 2008).

Several conservative treatment strategies have been recommended for patients suffering from GT, i.e., rest, shock-wave therapy, and corticosteroid injections (Brinks et al., 2011; Mellor et al., 2018; Ramon et al., 2020). However, to our best knowledge none of the previous treatment modalities promoted long lasting effects on patient reported function and/or pain (Brinks et al., 2011; Mellor et al., 2018; Ramon et al., 2020). Interestingly, low-load exercises performed daily combined with patient education have been observed to be superior to corticosteroid injections and a “wait and see” approach on patient reported global improvement and hip pain intensity 1 year after the intervention in patients with GT (Mellor et al., 2018). However, a high-frequent low-load exercise regimen to reduce symptoms is in sharp contrast to the literature on other lower limb tendinopathies (Kongsgaard et al., 2009; O'Neill et al., 2015). In clinical practice it is consistently reported that patients report severe pain exacerbations when moderate-to-high exercise loads are applied and/or the total training volume is progressed too fast. Therefore, low-load exercise regimens are highly warranted for this patient population.

Low-load exercises with concurrent restriction of the blood flow by means of a pneumatic cuff placed on the proximal part of the exercising extremity (LL-BFR) has consistently demonstrated promotion of skeletal muscle hypertrophy and increase strength in both patients and healthy individuals (Hughes et al., 2017; Lambert et al., 2018). Additionally, LL-BFR has been observed to increase muscle strength to the same extent as heavy load resistance strength training (Grønfeldt et al., 2020).

Recent studies indicate that LL-BFR may improve tendon morphology in both patients and healthy individuals (Sata, 2005; Centner et al., 2019; Skovlund et al., 2020). That is, LL-BFR has demonstrated to increase blood lactate level (Reeves et al., 2006; Manini et al., 2012) and stimulate growth hormone secretion, both suggested to contribute to tendon wound healing by upregulating the collagen synthesis (Klein et al., 2001; Yalamanchi et al., 2004; Boesen et al., 2013; Ilett et al., 2019).

To date, no studies have investigated the feasibility of LL-BFR in patients suffering from GT. Interestingly, improvements on skeletal muscle hypertrophy and strength in muscle groups proximal to the cuff have been demonstrated (Abe et al., 2005; Yasuda et al., 2010, 2011; Bowman et al., 2019). Additionally, it has been suggested that LL-BFR may trigger exercise-induced hypoalgesia comparable to levels seen after high intensity exercise (Hughes and Patterson, 2019). Thus, LL-BFR appears to be a relevant exercise treatment for this particular patient population.

The aim of this study was to examine the feasibility of LL-BFR combined with patient education for patients with GT in terms of adherence, dropouts, and adverse events. A secondary purpose was to evaluate changes in lateral hip pain, patient-reported outcomes, functional performance, and hip and knee muscle strength after an 8-week LL-BFR intervention.

## Materials and Methods

### Design

Feasibility study.

### Setting

Patients were referred to the study by orthopedic specialists and physiotherapists working in orthopedic outpatient clinics at three public hospitals (Horsens Regional Hospital, Aarhus University Hospital, Silkeborg Regional Hospital).

The intervention and testing took place at two hospitals only (Horsens Regional Hospital and Aarhus University Hospital). The intervention was conducted by two physiotherapists, while one of these (MH) conducted all tests.

### Participants

Inclusion criteria were (1) subjective complaints of lateral hip pain, (2) palpable tenderness or pain at the insertion point of the gluteus medius/minimus tendon at the greater trochanter, (3) a positive Single Leg Stance test (SLS), by reproduction of know lateral hip pain within 30 s of one leg stance, (4) lateral hip pain provoked by the FADER (Flexion Adduction External Rotation) and/or the FADER-R (Flexion Adduction External Rotation with resisted isometric internal rotation) test, (5) age 18–75 years and (6) ability to read and understand Danish. The diagnosis was based on the clinical tests described by Grimaldi et al. (2017). Exclusion criteria were (1) corticosteroid injection in the affected hip within the last 6 weeks prior to the intervention (2) any prior surgery in the affected hip (3) unregulated hypertension (≥ 180 mmHg/ ≥ 110 mmHg) (4) complaints or clinical signs of bilateral GT (5) MRI or X-ray verified osteoarthritis or (6) pregnancy. See Supplementary Material no. 1 for the clinical tests.

### Ethical Considerations

The study was conducted in accordance with the Declaration of Helsinki. The study was presented to the ethics committee of Central Region Denmark, who decided that no formal ethical approval was required (record number: 1-10-72-181-20). The Danish Data Protection Agency (record number: 1-16-02-548-20) approved the study and all patients gave written informed consent prior to inclusion.

### Intervention

The intervention had an overall duration of 8 weeks and consisted of exercises and patient education. Once a week, exercise sessions were performed at the hospital under supervision of the primary investigator (MH) or a trained physiotherapist (LCUR). Remaining exercises sessions were performed at home without supervision, yielding a total of eight supervised sessions and 48 sessions at home.

At baseline and at eight-week follow-up, patient-reported outcomes, two tests of physical function, isometric hip and knee muscle strength tests were completed.

### Exercise Program

The exercises chosen in this study were inspired by a previous study by Mellor et al. consisting of four to six exercises per session (Mellor et al., 2018). However, due to the duration of rest between sets and exercises using LL-BFR, only four exercises per session were chosen for this study. Static abduction, sidestepping, glute bridging, and bodyweight squats were chosen, as these exercises included functional retraining, strengthening of the hip and thigh muscles and control of adduction during function as proposed by Mellor et al. (2018).

**Figure 1:**
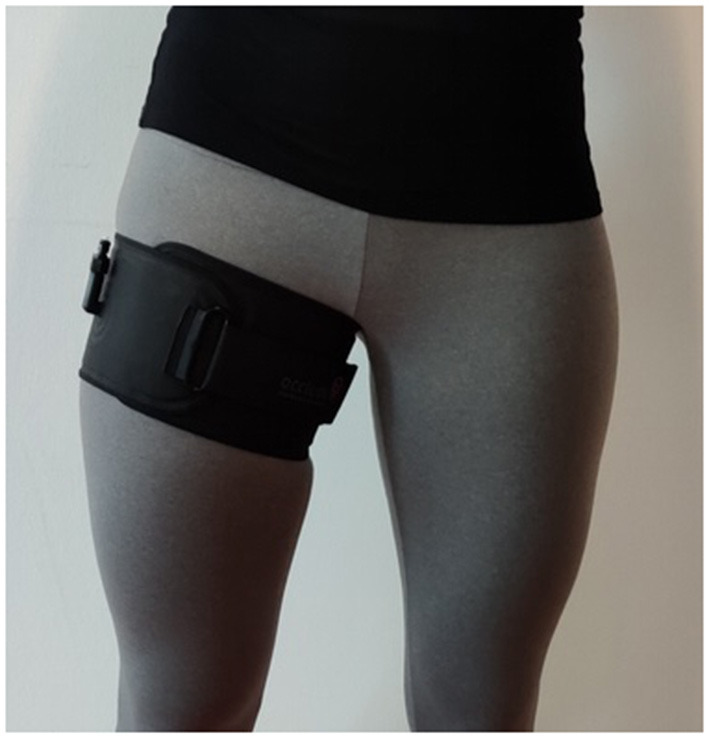
Cuff placement.

**Figure 2:**
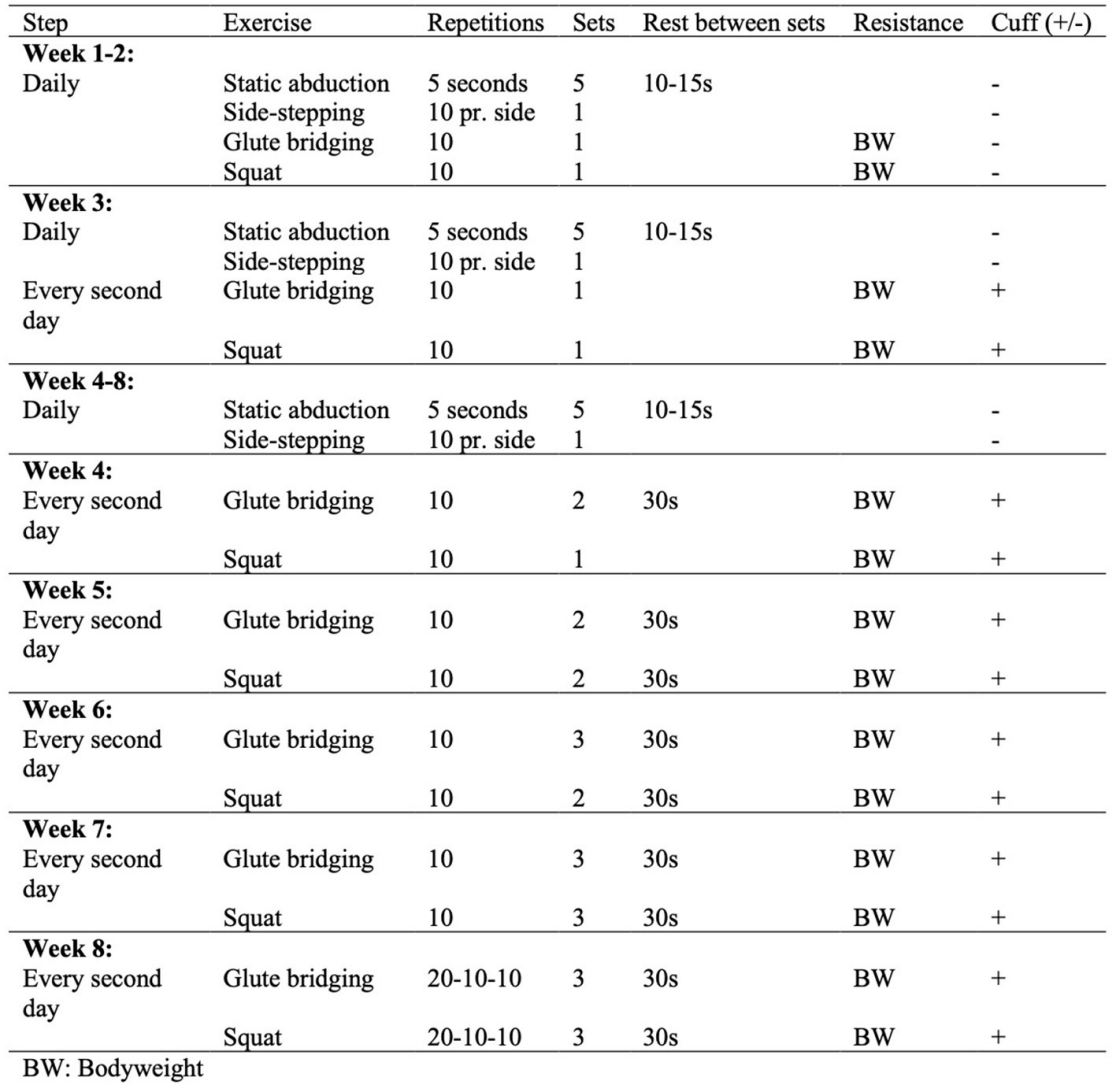
Exercise program.

Week 1 and 2, each training session consisted of the following: 5 × 5 s of standing static abduction, side-stepping; 10 steps to each side, 10 repetitions of glute bridging and 10 repetitions of squats. The exercises were to be performed once a day with bodyweight only. From week 3 to 8, static abduction and side-stepping were continuously performed daily. Glute bridging and squats were only performed every second day, and these two exercises were exclusively performed with the application of an 11.7 cm wide pneumatic BFR nylon cuff (Occlude Aps, Denmark) around the affected leg (Figure 1).Cuff pressure was 60% of the pressure required to fully restrict blood flow to the exercising limb. Given the relatively low volume in regards of repetitions of the BFR exercises from week 3 to 7, the restriction time was much shorter than suggested by Patterson et al. (5–10 min per exercise) (Patterson et al., 2019). That is, the squat and the bridging exercise combined, would until week 7 only last ~4 min. Thus, in order to reach the proposed restriction time, both the rest between exercises and a 30-s extra period after completion of the exercises were carried out. Given the increased number of repetitions, the participants released the pressure in the pause between the exercises in week 7 and 8. A LL-BFR session progressed from ~15 min in week 3 to ~25 min in the final 2 weeks. Repetitions in double leg bridging, and double leg squats were alternately increased by 10 repetitions every week until patients performed three sets of 20-10-10 repetitions. The full exercise programme with progressions is presented in Figure 2. The whole intervention is described according to the Template for Intervention Description and Replication (TIDieR) checklist (Hoffmann et al., 2014) in Supplementary Material No. 2. In case of illness during the intervention-period, which would result in absence from a supervised session, the progression for the following week and the patient education was managed by a telephone call. Throughout the intervention-period, patients were required to complete a daily training diary. In addition to reporting the number of repetitions, patients reported their perceived hip-related pain on a Numerical Rating Scale (NRS: 0-10) for every exercise and scored their perceived rating of exertion (RPE: 0-10) for the entire session. Diaries for the previous week were collected at the supervised sessions.

### Patient Education

At baseline testing, all patients received written information on the anatomy of the gluteus muscles, common GT-symptoms, pain management, load management, appropriate movement patterns, and resting positions. Patients were encouraged to avoid hip adduction across midline, prolonged single leg stance, lying on the affected side, and to place a pillow between the knees during sleep, and rest on the non-affected side to reduce tension and compression of the gluteus medius muscle and tendon. During supervised sessions, patients were continuously educated to manage their GT-symptoms according to the written information received at baseline, and time was taken to verbally instruct and ensure the understanding of the information regarding tendon care and load and pain management.

### Measurement of Limp Occlusion Pressure (LOP)

To individualize the cuff pressure during the exercise session, measurement of the pressure required to fully restrict blood flow to the affected lower limb [limp occlusion pressure (LOP)] was determined prior to week 3. A rigid nylon cuff (Occlude Aps, Denmark) with a removable manometer was fitted around the proximal thigh on the affected leg. The posterior tibial artery was located to detect the auscultatory pulse using a vascular doppler probe (EDAN SD3, China). The cuff was gradually inflated until the auscultatory pulse was undetectable as described previously elsewhere (Jørgensen et al., 2020). During LL-BFR exercises a cuff pressure corresponding to 60% LOP was applied and remained constant throughout the entire intervention period. As LOP is affected by body position (Sieljacks et al., 2018), LOP was measured in both seated and supine reflecting the body position of the torso during mini-squats and glute bridging. Subsequently, patients were carefully instructed to apply and inflate the cuff correctly, and further to regulate the pressure between the exercises according to either the mini-squats or glute bridging, without totally deflating the cuff.

### Outcome Measures

#### Feasibility

Adherence was measured as the proportion of exercise sessions completed in relation to the planned exercise sessions. Acceptable adherence was a priori set as a patient completing ≥80% of the planned sessions. Drop-out was defined as any reason for failure to continue the intervention and/or complete follow-up tests. Reasons for dropout was noted. A drop-out rate of 15% was considered acceptable. Adverse events were defined as any unexpected pain sensation, musculoskeletal injury, or cancellation of training sessions due to pain associated with the LL-BFR.

#### Descriptive Measurements

Self-reported bodyweight and height were collected, and body mass index was calculated (kg/m^2^). Further, self-reported duration of pain, educational level, marital status, and children (yes/no) were collected.

#### Patient-Reported Outcomes

Following questionnaires were completed at baseline and follow-up:

(i) The Victorian Institute of Sport Assessment-Gluteal questionnaire (VISA-G) is validated for measuring the severity of disability associated with GT (Fearon et al., 2015). VISA-G is comprised of eight questions and quantifies pain related to GT during loading (score range 0–100), where a higher score indicates less disability and pain.

(ii) The European Quality of Life −5 Dimensions Visual Analogue Score (EQ5D-VAS) is a vertical visual analogue scale (0–100, worst-best) on which the responder scores his/her perception of their overall health at a given day (Balestroni and Bertolotti, 2012).

(iii) The Oxford Hip Score (OHS) is a 12-item patient-reported outcome developed and validated to assess hip pain and function in patients undergoing total hip replacement with a composite score ranging from 0 (worst) to 48 points (best) (Wylde et al., 2005).

iv) The Copenhagen Hip And Groin Outcome Score (HAGOS), is a valid and reliable patient-reported outcome for hip and groin pain in young to middle-aged individuals [score range: 0 (worst) – 100 (best)] (Thorborg et al., 2011). The HAGOS score consists of six separate subscales (pain, symptoms, physical function in daily living, physical function in sport and recreation, participation in physical activities, and hip and/or groin related quality of life).

Pain during exercise in the 8 weeks on a Numerical Rating Scale (NRS) from 0 to 10 (0 = no pain, 10 = worst imaginable pain) was measured. Scores ≤5 were considered acceptable (Mellor et al., 2016).

Further, at follow-up, patients filled out the Global Rating of Change score (GRoC). GRoC consists of a 11-point scale, where the patient rates the perceived overall change of the hip condition from “very much better” to “very much worse” (Kamper et al., 2009). Responses on GRoC were considered successful if patients scored “moderately better” to “very much better.” Global improvement was measured as the percentage of successful reports.

#### Performance-Based Outcomes

Performance-based function was tested using a 30-s Chair-Stand Test (30s-CST) and a Stair Climb Test (SCT).

**The 30s-CST** is a test of functional capacity (Alcazar et al., 2020). The patient is seated in a chair with a seat height of 46 cm, and with their feet on the floor, placed shoulder-width apart with the arms crossed across the chest. During the test the patient moves from the sitting position to standing position with the hips at least in neutral position. The tester demonstrates the test prior to the patient's attempt. The patient completes as many stands and sits as possible in 30 s. The number of sits to stand completed in 30 s was recorded by MH.

**Stair ascending (SCT)** is a low-cost test used to estimate muscle power output (Cormie et al., 2011) (se calculation below). In this study, the patients were instructed to ascend a flight of stairs as fast as possible comprising of 11 steps with a total distance of 7.46 meters. A timer was started at the tester's command at the base of a staircase and stopped when the patient reached the top of the staircase with both feet on the last step. This time in seconds was then transformed into watts by the following formula used in (Novoa et al., 2015):


(1) $$\begin{array}{l} \frac{bodyweight\left(kg\right)\times 9.8\times altitude\;ascended\;\left(m\right)}{time\;taken\;\left(s\right)}=Power\;\left(W\right) \end{array}$$

#### Muscle Strength

Maximal voluntary isometric contraction (MVIC) was measured using a handheld dynamometer (HHD) (JTech Commander PowerTrack Muscle Dynamometer MMT, USA).

**Isometric hip abduction (MVIC HA)** was performed following a previously described test protocol (Kemp et al., 2013), i.e. with the patient lying supine on an examination bed while the patient's foot was resting on the examination bed. The HHD was placed 5 cm above the lateral malleolus. With the non-testing leg fixated with a belt around the table, the patient was instructed to push as forcefully as possible into the HHD while maintaining full hip and knee extension. The patient was given 3 trials on each limb.

**Isometric hip extension (MVIC HE)** followed the protocol in the study by Kemp et al. (2013). Briefly, MVIC HE was performed with the patient lying prone on an examination bed, the examined knee flexed 90° and the HHD placed on the heel. The patient was instructed to push as forcefully as possible, upwards into the HHD, trying to lift the knee and thigh free of the surface of the examination bed. The patients were given 3 trials on each limb.

**Isometric knee extension (MVIC KE)** was measured with the patient seated with both hip and knees positioned in 90° flexion, with the feet hanging over the edge of the examination bed. The HHD was placed 5 cm above the lateral malleolus, anterior to the tibia, on a shin guard strapped around the patient's shin. This was a slight modification to the protocol (Koblbauer et al., 2011), which used the medial malleolus as fixation point instead. To keep the HHD in position, a strap was attached to the examination bed, long enough to keep the patient's knee flexed at 90° during the test. The patient was instructed to kick/push as hard as possible into the HHD. Three trials on each limb were given.

### Statistical Analysis

Baseline characteristics were described using numbers (n), proportions (%) for categorical data, and mean and standard deviations (SD) for continuous data if the values were normally distributed. Otherwise, median and interquartile range (25th−75th percentiles) were presented.

Adherence was calculated as the proportion of completed exercise sessions for both the overall number of sessions and for the LL-BFR-sessions alone. Formula used to calculate the adherence:


(2) $$\begin{array}{l} \begin{array}{c} \begin{array}{l} \frac{\left(\frac{Total\;exercise\;sessions\;completed}{Total\;weeks\;completed}\right)}{Number\;of\;weekly\;exercise\;sessions\;scheduled}\times 100\;\\ \;=Adherence\;(\%) \end{array} \end{array} \end{array}$$

Changes in secondary outcome measures from baseline to follow-up were evaluated by using paired t-tests given the data were normally distributed, otherwise a Wilcoxon signed-rank test was used. Level of significance was set at 5%. Stata 16.0 (Statacorp, Texas, TX USA) was used for the statistical analysis. Proportion (%) of successful global improvements was presented using descriptive statistics.

## Results

### Eligible Patients

The study was conducted from October 2020 to April 2021. Sixteen females were included, and 14 completed the intervention. Baseline characteristics are presented in Table 1. Two patients had their intervention period extended from 8–9 weeks (one due to COVID-19, and one due to excessive hip-related pain at the time of initiation of LL-BFR).

**Table 1:** Baseline characteristics (*n* = 16).

**Variable**	
Sex, women, *n*	16
Age, years, median (IQR)	51 (45–60)
Height, cm, median (IQR)^*^	166 (164–169) (*n* = 15)
Weight, kg, median (IQR)^*^	70 (65–86) (*n* = 15)
Body Mass Index, kg/m^2^, median (IQR)	26.96 (23.59–30.46) (*n* = 15)
**Affected side**, ***n*** **(%)**	
Left	8 (50%)
Right	8 (50%)
**Occupation status**, ***n*** **(%)**	
Employed	12 (75)
Unemployed	1 (6.25)
Incapacity benefit	1 (6.25)
Retired	2 (12.5)
**Children**	
Yes	15 (93.75)
**Pain duration**, ***n*** **(%)**	
<2 months	0
2–6 months	2 (12.5)
7–12 months	3 (18.75)
> 12 months	11 (68.75)
NRS pain, 0–10, mean (SD)	5.43 (1.3)
Cuff-pressure (mmHg), mean (SD)	Seated: 131 (19.5)
	Supine: 116 (24.1)

*IQR, Inter Quartile Range; SD, standard deviation; NRS, Numeric rating scale*.

* *Weight and height were missing for one patient, who was a dropout*.

### Feasibility and Adherence

The mean (SD) adherence to all exercise sessions was 96% (5.76), with the patient with the lowest adherence displaying an adherence of 79%. The mean (SD) adherence to the LL-BFR-sessions only was 94% (8.52), with the patient with the lowest adherence achieving an adherence of 72%. One patient dropped out due to illness prior to the initiation of the LL-BFR. Another withdrew due to excessive pain during LL-BFR at home.

### Adverse Events

One adverse event, leading to a drop out, was registered during the intervention. The patient experienced a sudden and ongoing pain sensation during a LL-BFR session. The intensity of the pain was reported as 8-9 NRS. The pain was located directly beneath the LL-BFR-cuff and radiating throughout the leg alongside the affected leg. The patient counselled her general practitioner who advised her to stop her participation in the study. Two weeks later, the patient reported to have resumed her daily exercise sessions without the LL-BFR-cuff and did not experience pain with exercise.

### Patient-Reported Outcomes

Patient-reported hip pain, function, and quality of life from baseline to follow-up are presented in Table 2. Overall mean lateral hip pain (NRS) decreased significantly. Both VISA-G, EQ-5D-VAS, OHS and the majority of the subscales in HAGOS (5 out of 6) improved significantly. Nine out of 14 patients (64%) reported successful improvements on the GRoC. Mean (SD) hip pain during exercise throughout the intervention-period was 2.20 (1.43).

**Table 2:** Patient-reported outcomes (*n* = 14).

**Outcome**	**Baseline**	**Follow-up**	**Mean change**	***p***
	**mean [95% CI]**	**mean [95% CI]**	**[95% CI]**	
Pain (NRS, 0–10)	5.43 [4.65;6.20]	2.71 [1.82;3.60]	−2.71 [−3.71; −1.72]	<0.001
VISA-G (0–100)	56.57 [50.26;62.89]	66.57 [57.04;76.10]	10 [0.20;19.80]	0.046
EQ5D-VAS (0–100)	68.36 [59.69;77.02]	80 [72.84;87.16]	11.64 [3.33;19.96]	0.009
Oxford hip score (0–48)	29 [24.75;33.25]	36.6 [32.86;40.37]	7.6 [4.57;10.66]	<0.001
**HAGOS (0–100)**				
Symptoms	49.75 [43.00;56.49]	69.90 [58.85;80.95]	20.15 [9.47;30.83]	0.001
Pain	53.39 [44.47;62.32]	69.82 [58.99;80.64]	16.43 [6.61;26.25]	0.003
ADL	55.71 [46.74;64.69]	70 [55.59;84.41]	14.29 [3.73;24.84]	0.011
Sports/Rec	40.85 [30.19;51.49]	59.82 [44.16;75.49]	18.97 [6.38;31.56]	0.006
PA	33.93 [19.38;48.48]	41.07 [22.20;59.94]	7.14 [-10.47;24.76]	0.397
QOL	31.43 [25.39;37.47]	45.00 [31.94;58.06]	13.57 [1.78;25.36]	0.027

*VISA-G, Victorian Institute of Sport Assessment-Gluteal; EQ5D-VAS, European Quality of Life - 5 Dimensions - Visual Analogue Scale; NRS, Numeric rating scale; HAGOS, Copenhagen Hip and Groin Outcome Score; ADL, Function in daily living; Sports/Rec, Function in sport and recreation; PA, Participation in Physical Activities; QOL, Hip and/or groin-related Quality of Life*.

### Performance-Based Outcomes

The mean number of repetitions in the 30s-CST as well as the mean power output in the SCT increased significantly from baseline to follow-up (Table 3).

**Table 3:** Performance-based outcomes and isometric muscle strength (*n* = 14).

**Outcome**	**Baseline (mean [95% CI])**	**Follow-up (mean [95% CI])**	**Mean change (baseline-Follow-up) mean [95% CI]**	***p***	**Diff. of change between legs (Follow-up), mean [95% CI]**	***p***
30s-CST, no of reps	14.6 [12.2;17.1]	19.4 [16.3;22.4]	4.7 [2.9;6.5]	<0.001		
SCT, Watt	282.61 [247.16;318.05]	334.99 [289.88;380.12]	52.39 [12.62;92.16]	0.014		
**MVIC, Hip abduction, Nm/kg,**						
Affected leg	0.81 [0.67;0.96]	1.02 [0.87;1.17]	0.21 [0.11;0.31]	<0.001	0.10 [0.03;0.18]	0.012
Non-affected-leg	0.99 [0.85;1.12]	1.09 [0.95;1.23]	0.10 [-0.02;0.23]	0.083		
**MVIC, Hip, extension, Nm/kg**						
Affected-leg	0.43 [0.34;0.53]	0.70 [0.55;0.86]	0.27 [0.15;0.39]	<0.001	0.08 [-0.01;0.16]	0.067
Non-affected-leg	0.53 [0.40;0.65]	0.72 [0.57;0.86]	0.19 [0.13;0.24]	<0.001		
**MVIC, Knee extension, Nm/kg**						
Affected—leg	1.05 [0.80;1.30]	1.26 [0.97;1.56]	0.21 [0.02;0.40]	0.031	0.10 [-0.01;0.20]	0.061
Non-affected-leg	1.16 [0.93;1.38]	1.27 [1.00;1.54]	0.11 [-0.02;0.25]	0.085		

*30s-STS, 30 second sit-to-stand; SCT, Stair Climb Test; MVIC, Maximal Voluntary Isometric Contraction*.

### Isometric Muscle Strength

Isometric muscle strength outcomes are presented in Table 3. Significant improvements of isometric strength were seen in the affected leg in both MVIC HA and MVIC KE, and in both legs in MVIC HE. The difference in strength change between the affected and unaffected leg was only significant in MVIC HA.

## Discussion

The main finding of this study was that 8 weeks of LL-BFR and patient education was feasible in the included female population suffering from GT. In general, adherence to the exercise intervention was high (96%) and the drop-out rate low (13%). Only one patient dropped out due to LL-BFR related pain exacerbation. Additionally, LL-BFR was performed without an augmented pain sensation. At follow-up, a clinically relevant reduction in lateral hip pain and improvements of both patient-reported outcomes, functional capacity and isometric muscle strength were seen.

### Feasibility Outcomes

In the present study we observed an exercise adherence corresponding to 96%, which is higher than the adherence previously reported with daily exercise sessions planned for patients suffering from GT (Ganderton et al., 2018; Mellor et al., 2018). Mellor et al. reported a mean (SD) adherence at 88.8% (13.7) after 8 weeks of daily home-based/supervised low-load exercise intervention (Mellor et al., 2018). Ganderton et al. reported an adherence corresponding to 75.80% (23.49) for the intervention group after 12 weeks of a low-load exercise and 75.99% (25.35) for the control group, engaging in 12 weeks of a sham exercise involving primarily seated exercises with no external load (Ganderton et al., 2018). Recently, other homebased LL-BFR exercise protocols have emerged and demonstrated excellent adherence when applied in clinical populations (Kilgas et al., 2019; Petersson et al., 2020; Jørgensen and Mechlenburg, 2021). Both Petersson et al. (2020) and Jørgensen and Mechlenburg (2021) have reported adherence of 100% to a 5-week combined homebased and supervised BFR walking exercise protocol (three sessions/week, one of these supervised) in a patient suffering from knee OA and to a 12-week homebased LL-BFR intervention (exercise session every second day, supervision only during the first week) in a patient suffering from reactive arthritis, respectively (Petersson et al., 2020). In line with this, Kilgas et al. reported an adherence of 100% to a 4-week entirely homebased LL-BFR intervention (40 sessions in total) in a patient who had received a total knee arthroplasty (Kilgas et al., 2019). Thus, the adherence in this present study was consistent with previous studies, even though our study had more patients included.

To our knowledge, only a few smaller studies have investigated the feasibility of LL-BFR in clinical settings for patients with tendinopathies (Sata, 2005; Skovlund et al., 2020). A case series by Skovlund et al. investigated the feasibility of LL-BFR on patients with patellar tendinopathy (21). This study reported a 50% pain-reduction on NRS during a single-leg decline squat test, while the tendon vascularity was decreased by 31% after 3 weeks of low-load BFR resistance exercise (Skovlund et al., 2020). In line with these findings, a case report from 2005 observed reduced pain levels (without having reported the magnitude of this) and no thigh muscle atrophy after 3 weeks LL-BFR exercise in a patient showing patellar tendinitis (Sata, 2005). In both studies the adherence was comparable to the findings in the present study. Further, none of the studies reported any adverse events. However, both studies had few participants, and the exercise protocols consisted exclusively of supervised training sessions.

A concern regarding supervised exercise session in clinical populations is the limited flexibility in time, since both the patient and the supervisor has to have compatible timetables (Collado-Mateo et al., 2021). In this study, the supervised exercise sessions were performed at two sites to allow patients access to the supervised exercise session closer to either their home or worksite. Further, a session consisted of maximum four exercises (every second day from week 3) with no required exercise equipment beside the cuff, which was handed out at the hospitals at baseline. Additionally, in both the present study and the study by Mellor et al. patient education took place during the weekly, supervised sessions. This study design could enhance the patients understanding of their condition and expected benefit of the exercises (Collado-Mateo et al., 2021), resulting in a higher compliance to the protocol.

In the present study, one adverse event, leading to a drop out, was registered. There has been some discussion on the safety of LL-BFR, underreporting adverse events (Minniti et al., 2020) and the degree to which LL-BFR can cause serious muscle damage, such as exertional rhabdomyolysis (Wernbom et al., 2020). However, the patient reporting an adverse event in this study was not displaying symptoms related to rhabdomyolysis or other muscle damages, as the patient reported pain ease and continuation of daily exercise 2 weeks after the incident.

In general, LL-BFR seems to be a well-tolerated and a safe exercise modality in both healthy and clinical populations, when safety precautions concerning cuff application, cuff pressure, and time with blood flow restriction are taken (Hughes et al., 2017; Patterson et al., 2019).

### Patient-Reported Outcomes

Improvements in patient reported outcomes shown in this study were comparable to Mellor et al. and Ganderton et al., although our patient population was slightly younger and had a longer period of pain duration before inclusion (Ganderton et al., 2018; Mellor et al., 2018). Changes in HAGOS score after an intervention in GT patients has to our knowledge, not been reported before. Five of the six subscales on HAGOS showed significant improvements. Additionally, a mean change within the minimal important change (MIC) of 10-15 points were seen in these five subscales (Thorborg et al., 2011). Even though the patients experienced less pain and better functioning, the subscale “participation in physical activities” (PA) only improved by 7 points. However, 14 patients had a duration of symptoms for at least 7 months suggesting that fear-avoidance or habits may have changed their approach to physical activities. Further, the PA subscale only consist of two items which make it a challenge to achieve a minimal important change.

### Performance-Based Outcomes and Isometric Muscle Strength

In the present study, hip abduction strength measured at follow-up was comparable to the abduction strength in the exercising group in the study by Mellor et al. at 8 weeks follow-up (Mellor et al., 2018). Further, we found that mean hip extension peak torque increased for both legs and knee extension mean peak torque increased on the affected leg. Interestingly, the strength deficits observed at baseline between the affected and non-affected leg was minimized at follow-up. These gains in strength are reflected in the functional capacity tests, where the number of repetitions in the 30s-CST and the power output used in the SCT improved, indicating better function. Previous research by Fearon et al., suggested pain to be the main driver to activity limitation following GT (Fearon et al., 2017). Hence, the clinically relevant reduction in pain observed in the present study may have contributed to improvements in functional performance and muscle strength.

### Adaptations Proximal to the Cuff

Intuitively, the muscular adaptations to LL-BFR are expected to occur distal to the cuff. Nevertheless, Hedt et al. focused a review on proximal muscle responses to BFR training and suggested a potential benefit of the tissue directly proximal to the occlusion site (Hedt et al., 2022). The mechanisms for proximal benefits of BFR requires more research. However, the authors suggested that increased muscle activation of the muscle proximal to the cuff due to downstream fatigue, mechanotransduction signaling (due to muscle cell swelling), metabolite signaling during release of cuff pressure, and systemic anabolic signaling (i.e. insulin growth factor-1, growth hormone) as potential mediators of the adaptations proximal to the occlusion site. Even though studies on proximal effects after BFR training primarily have focused on the upper extremity (Hedt et al., 2022), a RCT study by Bowman et al. has, in line with the present study, observed increased isometric hip abduction muscle strength following 6 weeks of unilateral LL-BFR training (Bowman et al., 2019).

## Limitations

Some limitations to the present study must addressed. Given the lack of control group and a small sample-size, effect of the intervention, i.e., LL-BFR exercise and patient education, and the outcomes, cannot be evaluated in this study.

Furthermore, we chose to include women only, as GT mainly affects women, which may negatively affect the external validity. Moreover, we did not register how many patients were screened for eligibility to this study and have no overview of the overall patient flow in the three referring orthopaedic outpatient clinics, hence the included patients might not represent a broader population of patients with GT. Demographics of our population is however, comparable to the women included in the studies by Grimaldi et al. (2017) and Mellor et al. (2018).

No imaging modalities were used to diagnose tendinopathy in the present study. However, the clinical tests used in this study, have been shown by Grimaldi et al. to be useful in diagnosing GT (Grimaldi et al., 2017).

A recent position stand by Patterson et al. (2019) proposed a guideline for applying LL-BFR training. Most of the recommendations are met in the present study in terms of cuff pressure and measurement of LOP, restriction time, rest time and frequency. However, the loading intensity and number of repetitions did not meet the guideline (Patterson et al., 2019), as Patterson et al. suggested using loading intensities of 20–40% of 1 repetition maximum (1RM) and a total of 75 repetitions. In the present study, exercises were performed with bodyweight only, thus, it is doubtful whether the proposed load has been met. Further, the maximal number of repetitions in an exercise reached 40 repetitions in 3 sets. The lower volume might be reflected in the patients perceived exertion-levels, as RPE was observed to be 2.25 (1.48). However, based on clinical experiences with patients suffering from GT and considering the proposed treatment strategies for controlling high tensile loads in patients with gluteal tendinopathy outlined by Grimaldi et al., an increased intensity of the exercises seems contraindicated (Grimaldi and Fearon, 2015). Thus, a low-load exercise protocol with slowly progression in exercise volume during the exercise period was considered viable. Another limitation related to the LL-BFR was the posture of which the patients had during the measurement of LOP. Hughes et al. have shown that LOP in the standing position is significantly higher than in a sitting position (Hughes et al., 2018). Since the patients in this study were sitting during the measurement of LOP for the mini-squats, the pressure in the cuff during the stand phase of the squat might have been lower than 60%.

## Conclusion

The present study demonstrated that our exercise protocol using LL-BFR, and patient education was safe and feasible in female patients suffering from GT. Despite a low dropout, one adverse event occurred, which confirms the necessity for regular monitoring of patients engaging in LL-BFR. Nevertheless, patients reported a clinically relevant pain reduction, improved patient reported outcomes and increased physical performance. Additional research is highly needed in terms of determining effects of LL-BFR in GT. As proposed by Ganderton et al. (2018) and Mellor et al. (2018) the effectiveness of the patient education might be underrated. Hence, RCT studies addressing the impact of patient education alone, as well as LL-BFR alone on both functional and patient-reported outcomes in GT are warranted.

## Data Availability Statement

The original contributions presented in the study are included in the article/Supplementary Material, further inquiries can be directed to the corresponding authors.

## Ethics Statement

Ethical review and approval was not required for the study on human participants in accordance with the local legislation and institutional requirements. The patients/participants provided their written informed consent to participate in this study.

## Author Contributions

NR, IM, MB, and SJ: contribution to conception. MB, SJ, and MH: design of the study. LT: performed the supervised exercise sessions at Aarhus University Hospital. MH: performed the supervised exercise sessions at Horsens Regional Hospital, handled the statistical analysis, and wrote the draft of the manuscript. All authors: reviewing and editing of manuscript.

## Conflict of Interest

The authors declare that the research was conducted in the absence of any commercial or financial relationships that could be construed as a potential conflict of interest.

## Publisher's Note

All claims expressed in this article are solely those of the authors and do not necessarily represent those of their affiliated organizations, or those of the publisher, the editors and the reviewers. Any product that may be evaluated in this article, or claim that may be made by its manufacturer, is not guaranteed or endorsed by the publisher.
